# Case Report: Tislelizumab-induced insulin-dependent diabetes mellitus: a case report and literature review

**DOI:** 10.3389/fphar.2025.1499796

**Published:** 2025-07-23

**Authors:** Panpan Ji, Yahui Zhang, Wen Zhang, Bing Leng, Ruifang Nie, Chengwu Shen, Hengcai Yu

**Affiliations:** ^1^Department of Pharmacy, Shandong Provincial Hospital Affiliated to Shandong First Medical University, Jinan, China; ^2^Department of Pharmacy, Jinan Beicheng Hospital, Jinan, China

**Keywords:** tislelizumab, diabetes, PD-1 inhibitors, immune checkpoint inhibitors, adverse event

## Abstract

Tislelizumab is approved for cancer immunotherapy. Tislelizumab-induced insulin-dependent diabetes mellitus (IDDM) is infrequent, but life-threatening; to date, no reports have been published in English. The study aims to analyze the adverse effect. A case of tislelizumab-induced IDDM was reported. The study also reviewed published cases of developing IDDM after using tislelizumab, by systematic search of PubMed, CNKI, WANFANG and VIP (the latter three, Chinese article databases). Seventeen patients (including ours) were included. The mean age was 65.7 years and 73% (11/15) were male. IDDM usually appeared after 8.4 cycles. The mean random glycemia was 35.9 mmol/L, however, the hemoglobin A1c was only 8.5%. Eighty percent (12/15) presented with diabetic ketoacidosis (DKA). One hundred percent showed inappropriately low C-peptide (15/15) and undetectable autoantibodies (14/14). Insulin was immediately administered to all patients and 50% (8/16) had relatively stable glycemic control lastly. Similar to previous reports, tislelizumab-induced IDDM is characterized by a more rapid progression to severe insulin deficiency, frequently with DKA. However, unlike previous ones, islet autoantibodies were absent in all cases, possibly because of racial differences. These findings offer valuable safety warnings and allow doctors to identify and treat tislelizumab-induced IDDM timely.

## 1 Introduction

In cancer immunotherapy, immune checkpoint inhibitors (ICIs) suppress the physiological blocks of immune responses, thereby activating T cells and leading to kill tumor cells (Kennedy and Salama, 2020). ICIs consist of antibodies targeting cytotoxic T lymphocyte antigen-4 (CTLA-4), programmed cell death protein 1 (PD-1) and programmed death-ligand 1 (PD-L1) (Kennedy and Salama, 2020). Tislelizumab (Tevimbra, BeiGene) is an anti-PD-1 antibody that selectively blocks interactions with the PD-1 receptor on T cells, which restores T cell activation and proliferation (Chinese Label, 2022). Tislelizumab was firstly approved for classical Hodgkin lymphoma (HL) by the National Medical Products Administration (NMPA) in China in December 2019 (NMPA Approval, 2019); the European Medicines Agency (EMA) in Europe followed suit in September 2023 (EMA Approval, 2023), and the U.S. Food and Drug Administration (USFDA) in March 2024 (FDA Approval, 2024). To date, NMPA has approved tislelizumab for the treatment of classical HL, urothelial carcinoma, non-small cell lung cancer (NSCLC), hepatocellular carcinoma, microsatellite instability-high solid tumors, esophageal squamous cell carcinoma, and nasopharyngeal carcinoma (Chinese Label, 2022).

Anti-PD-1 therapies are primarily associated with immune-related adverse events which manifest as rashes, pruritus, thyroiditis, diarrhea, hepatitis, and pneumonitis. Immunotherapy-induced insulin-dependent diabetes mellitus (IDDM) is infrequent, but life-threatening (Kennedy and Salama, 2020; Chang et al., 2019). Thus far, reports on anti-PD-1 antibody-induced IDDM only focused on pembrolizumab (Keytruda, Merck Sharp & Dohme) and nivolumab (Opdivo, Bristol-Myers Squibb) (Stamatouli et al., 2018; Byun et al., 2020; Kotwal et al., 2019; Clotman et al., 2018; Baden et al., 2018; Gauci et al., 2017; de Filette et al., 2019; Okamoto et al., 2016). To date, no reports of tislelizumab-related IDDM exist in English.

Herein, we report a case of tislelizumab-induced IDDM internationally for the first time and outline previous cases published in Chinese through a literature search (n = 17, including our case). Furthermore, we discussed the incidence, clinical presentation, therapy, risk factors, and potential pathogenic mechanisms of ICI- induced IDDM. It improved the data on ICI-induced IDDM and further provided references for doctors to identify and treat the adverse effect timely.

## 2 Case report

**FIGURE 1:**
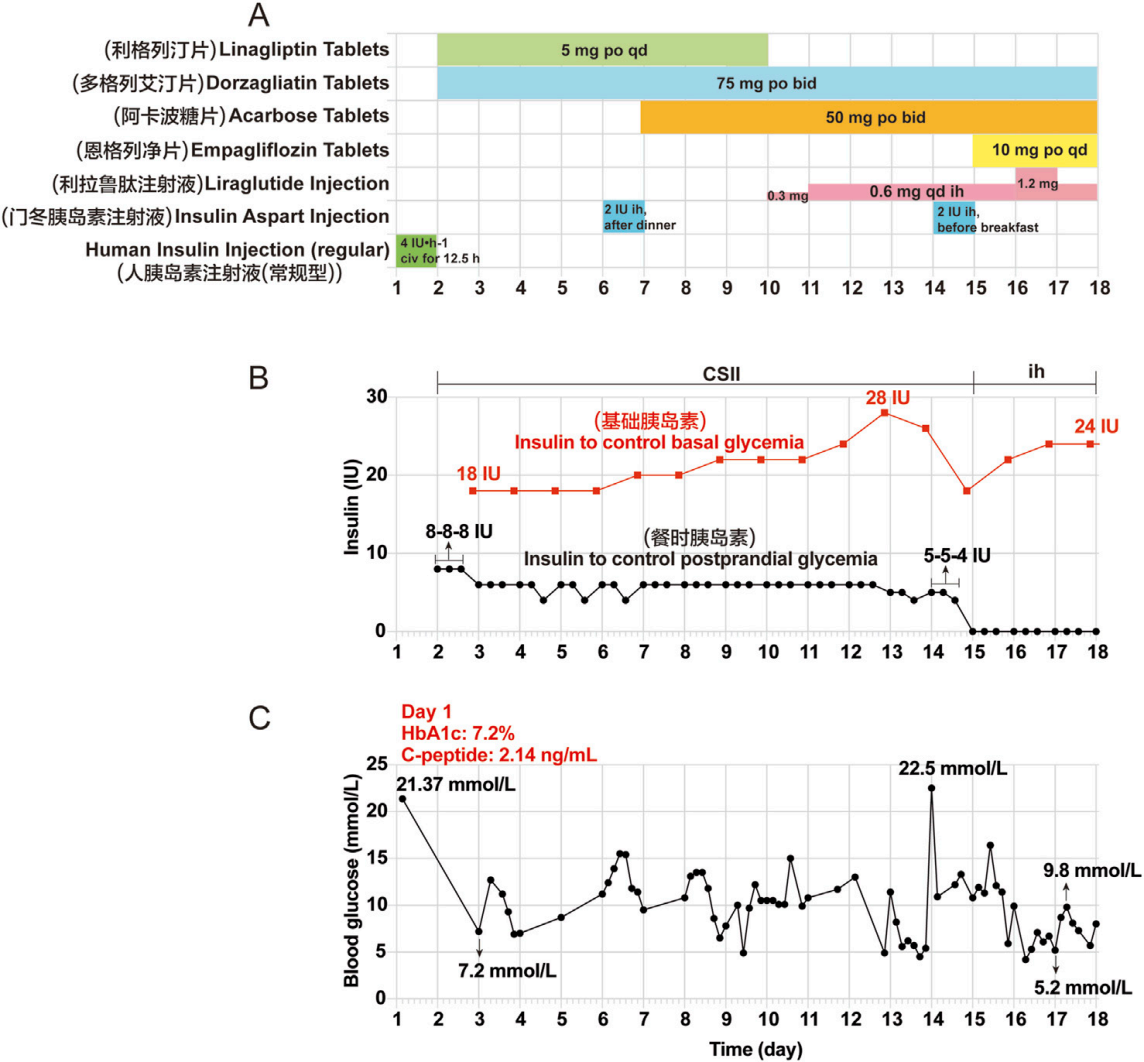
Clinical course of the patient after the first admission. **(A)** Other hypoglycemic drugs used during hospitalization from Day 1 (June 19) to Day 18 (July 6). **(B)** Insulin aspart injection was given to control basal and postprandial glycemia by CSII using an external pump from Day 2 to Day 14. Insulin degludec injection was given before bedtime by subcutaneous injections from Day 15 to Day 17. **(C)** Changes in blood glucose during hospitalization. The seven point-glycemia was monitored throughout the day: before and after breakfast, before and after lunch, before and after dinner, before bedtime. Among them, unmonitored glycemia are not showed. Civ: continuous intravenous pumping; po: per os; CSII: continuous subcutaneous insulin infusion; ih: hypodermic injection.

A 54-year-old Chinese man with a body mass index of 24.38 kg/m^2^, a history of hypertension and coronary heart disease, and no history of diabetes mellitus (DM), medicine, or food allergy had been receiving tislelizumab (200 mg once, once every 3 weeks) as treatment for renal transitional cell carcinoma since July 2023. After 11 cycles, the patient presented with dry mouth, polydipsia, and polyuria, without obvious induction. The patient’s random blood glucose level was 21.37 mmol/L on 19 June 2024 (Day 1). The relative complete biological investigations evidenced the following: urinary glucose: 4 + 2000 mg/dL; urinary ketone body: 1 + 10 mg/dL; serum C-peptide levels: 2.14 ng/mL (normal range, 1.1–4.4 ng/mL); hemoglobin A1c (HbA1c): 7.2% (normal range, 4%–6%); serum *β*-hydroxybutyrate: 0.578 mmol/L (normal range, 0–0.3 mmol/L); serum urea: 11.4 mmol/L (normal range, 3.1–8.0 mmol/L); serum creatinine: 110.90 μmol/L (normal range, 57–97 μmol/L); and serum Na^+^: 134.2 mmol/L (normal range, 137–147 mmol/L). The patient tested negative for all islet autoantibodies, including islet cell antibodies (ICA), insulin autoantibodies (IAA), anti-glutamic acid decarboxylase (GAD) antibody, anti-insulinoma-associated antigen-2 (IA-2) antibody, and anti-zinc transporter 8 (ZnT8) antibody. Based on these clinical data, the patient was diagnosed with “diabetes; ketosis” and treated with intravenous fluid resuscitation, continuous subcutaneous insulin infusion (CSII) using an insulin pump, and oral hypoglycemic drugs. The patient’s blood glucose levels improved, and he was discharged on 6 July 2024 (Day 18). He continued treatment with insulin degludec injection 24 iu *ih* qn, liraglutide injection 0.9 mg *ih* qd, acarbose tablets 50 mg *po* tid, and dorzagliatin tablets 75 mg *po* bid. The clinical course of the patient after this admission is shown in Figure 1.

**FIGURE 2:**
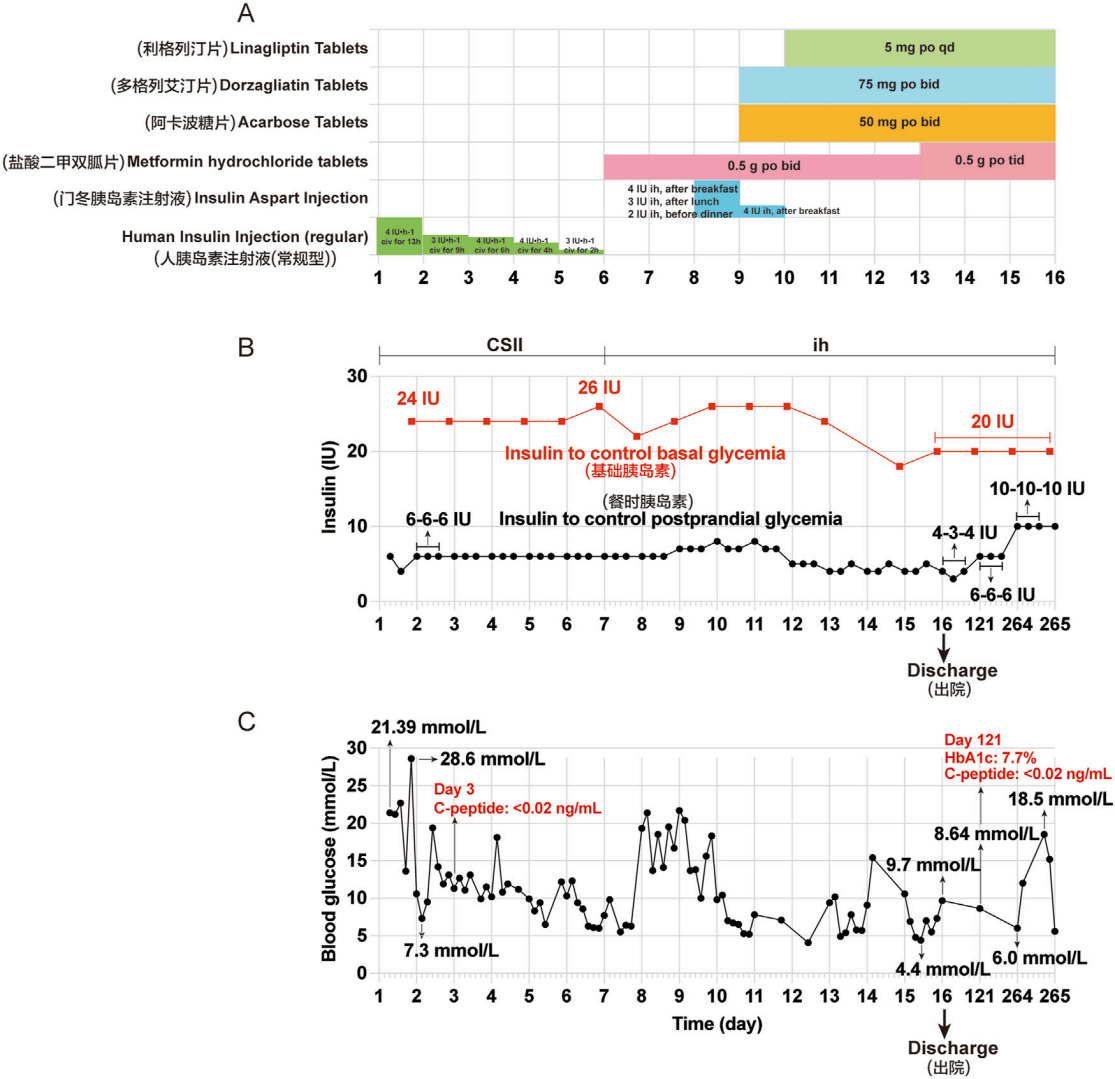
Clinical course of the patient during the second hospitalization and after discharge. **(A)** Other hypoglycemic drugs used during hospitalization from Day 1 (July 10) to Day 16 (July 25). **(B)** Insulin aspart injection was given to control basal and postprandial glycemia by continuous subcutaneous insulin infusion (CSII) using an external pump from Day 1 to Day 6. Insulin aspart injection was used before three meals and insulin degludec injection was used before bedtime by subcutaneous injections from Day 7 to Day 16, and at Day 121 and 264. **(C)** Changes in blood glucose during hospitalization and after discharge. The seven point-glycemia was monitored throughout the day: before and after breakfast, before and after lunch, before and after dinner, before bedtime. Among them, unmonitored glycemia are not showed. Civ: continuous intravenous pumping; po: per os; CSII: continuous subcutaneous insulin infusion; ih: hypodermic injection.

Owing to persistent symptoms of dry mouth, polydipsia, polyuria, along with hiccups, nausea, and vomiting for 4 days, the patient revisited our hospital on 10 July 2024 (Day 1). The initial laboratory results were as follows: random blood glucose: 21.39 mmol/L; serum *β*-hydroxybutyrate: 4.756 mmol/L; serum urea: 14.0 mmol/L; serum urea/creatinine: 125.98 (normal range, 20–100); serum complement C1q: 131.60 mg/L (normal range, 159–233 mg/L); serum Na^+^: 122.6 mmol/L; serum Cl^−^: 93.5 mmol/L (normal range, 99–110 mmol/L); serum CO_2_: 13.8 mmol/L (normal range, 22–29 mmol/L); serum P^3+^: 0.48 mmol/L (normal range, 0.85–1.51 mmol/L); serum lactate dehydrogenase: 110.0 U/L (normal range, 120–250 U/L); urinary glucose: 4 + 2000 mg/dL; and urinary ketone body: 3 + 80 mg/dL. The patient was readmitted to our hospital with DKA and received fluid resuscitation to eliminate ketones, correct the acid-base balance, electrolyte disturbances, and CSII using an insulin pump. Further testing revealed that his serum insulin levels dropped to below 0.40 μU/mL (normal range, 2.6–24.9 μU/mL) and serum C-peptide was nearly undetectable (<0.02 ng/mL) on 12 July 2024 (Day 3). These laboratory findings suggest a sudden deterioration in *β*-cell function, indicative of extreme hyperglycemia. Considering the lack of a history of DM and tislelizumab therapy, the patient was diagnosed with immunotherapy-induced IDDM with diabetic ketoacidosis (DKA). After the patient’s blood glucose levels improved, he was discharged on 25 July 2024 (D 16). He continued treatment with multiple daily injections of insulin (insulin degludec injection 20 IU *ih* qn; insulin aspart injection, 4 IU-3 IU–4 IU *ih*, before three meals a day) combined with oral hypoglycemic drugs (metformin hydrochloride tablets 0.5 g po tid; acarbose tablets 50 mg po tid; linagliptin tablets 5 mg po qd; dorzagliatin tablets 75 mg po qd) and discontinued tislelizumab. The patient was instructed to initiate a diabetic diet and continuous glucose monitoring. Follow-up results showed that the HbA1c was 7.7% and serum C-peptide was still nearly undetectable (<0.02 ng/mL) on 07 November 2024 (Day 121). Treatment with insulin combined with oral hypoglycemic drugs was continued, and blood glucose levels were generally controlled and fluctuated significantly. Tislelizumab immunotherapy remained discontinued. The clinical course of the patient during the second hospitalization and after discharge is shown in Figure 2.

Causality between tislelizumab and IDDM was evaluated using the Naranjo’s Causality Assessment Scale (Naranjo et al., 1981), and the results are shown in Table 1. A score of 7 indicated that tislelizumab was probably related with IDDM.

**TABLE 1:** Naranjo’s assessment scale of tislelizumab for IDDM.

Scoring items	Scoring criteria			Score of the current case	Scoring evidence
	Yes	No	Do not know		
1. Are there previous conclusive reports on this reaction?	+1	0	0	1	Tislelizumab-induced insulin-dependent diabetes has been mentioned in the drug label
2. Did the adverse event occur after the suspected drug was administered?	+2	−1	0	2	This reaction occurred after the administration of tislelizumab
3. Was the adverse reaction alleviated when the drug was discontinued or a specific antagonist was administered?	+1	0	0	1	The blood glucose levels improved after holding tislelizumab and administering insulin combined with oral hypoglycemic drugs
4. Did the adverse reaction reappear when the drug was readministered?	+2	−1	0	0	Tislelizumab was not readministered
5. Are there alternative causes (other than the drug) that could have independently caused the reaction?	−1	+2	0	2	No other cause can alone cause the insulin-dependent diabetes
6. Did the reaction reappear when a placebo was given?	−1	+1	0	0	The patient did not receive a placebo
7. Were the drug concentrations detected in the blood (or other fluids) known to be toxic?	+1	0	0	0	The concentrations of tirellizumabin in the blood (or other fluids) were not determined
8. Was the reaction more severe when the dose was increased, or less severe when the dose was decreased?	+1	0	0	0	The tislelizumab dose was not changed
9. Did the patient have a similar reaction to the same or similar drugs in any previous exposure?	+1	0	0	0	The patient denied previous exposure to the same or similar drugs
10. Was there any objective evidence to confirm the adverse event?	+1	0	0	1	The patients had extremely low C-peptide levels and significantly elevated blood glucose values with diabetic ketoacidosis
Total score				7	Probable

On assessment of causality, total score ≥9, definitely related; total score 5–8, probably related; total score 1–4, possiblely related; total score ≤0, doubtful.

## 3 Discussion

### 3.1 Literature review of tislelizumab-induced IDDM

Here, we reported a case of tislelizumab-induced IDDM at our hospital. In addition, systematic search of PubMed, CNKI, WANFANG, VIP (the latter three, Chinese article databases) using the terms “tislelizumab” or “immune checkpoint inhibitor” or “PD-1 inhibitor” and “diabetes” or “ketoacidosis” were performed. Articles published in English or Chinese from 1 August 2000, to 26 August 2024, were independently included by two researchers (Panpan Ji and Hengcai Yu).

We included all published case and series reports of tislelizumab-induced IDDM. Case reports, or series with severely incomplete clinical data (such as basic information, clinical manifestations, treatment, and outcomes), and duplicates were excluded. We provided an overview of all included studies (Kong et al., 2023; Zhang et al., 2022; Rui and Zhu, 2023; Zhang et al., 2024; Wang Y. Z. et al., 2024; Wang L. et al., 2024; Pang et al., 2024; Yang et al., 2023; Mei et al., 2024; Wang Y. et al., 2024; Han et al., 2023; Qiu et al., 2023).

Two researchers (Panpan Ji and Hengcai Yu) independently screened the published cases. The following items were extracted from each included study: author, year of publication, sex, age, primary diagnosis, tislelizumab dosage, combined drugs, cycles of treatment at diagnosis, clinical manifestations, random blood glucose levels at the first visit, HbA1c, C-peptide, pancreatic autoantibodies, DKA, other endocrine adverse events, treatment, and outcomes. We used the Common Terminology Criteria for Adverse Events 5.0 (CTCAE5.0) to identify the severity of adverse events of tislelizumab-indued IDDM (Freites-Martinez et al., 2021). When available, the supplementary data and appendices were explored methodically. Any discrepancies were discussed by all authors and resolved by consensus. Descriptive statistical analysis was performed on the extracted data.

We identified 13 articles that represented 18 cases. Two cases from one publication were excluded owing to severely incomplete clinical data (Lv et al., 2023). Seventeen patients with tislelizumab-induced IDDM were included in this study, including the current case report (Kong et al., 2023; Zhang et al., 2022; Rui and Zhu, 2023; Zhang et al., 2024; Wang Y. Z. et al., 2024; Wang L. et al., 2024; Pang et al., 2024; Yang et al., 2023; Mei et al., 2024; Wang Y. et al., 2024; Han et al., 2023; Qiu et al., 2023) (Table 2). Reassessment of causality between tislelizumab and IDDM in 17 cases revealed that 16 (94.1%) were probably related and one (5.9%) was definitely related, according to Naranjo’s causality assessment scale (Naranjo et al., 1981) (Table 2).

**TABLE 2:** Clinical characteristics and course of individual patients with tislelizumab-induced IDDM.

Patient	Authors, Y	Sex	Age (Y)	Primary diagnosis	Tislelizumab dosage	Combined drugs	Cycles of treatment at diagnosis	Clinical manifestations	Random blood glucose at the first visit (mmol/L)	HbA1c at the first visit (%)
1	Kong et al. (2023)	M	58	Lung cancer	NA	NA	18	Thirst, polydipsia, fatigue, nausea	34.85	8.3
2	Zhang et al. (2022)	F	75	Left ureteral epithelial carcinoma	200 mg once	NA	5	Dry mouth, fatigue	26.71	8.5
3	Rui and Zhu (2023)	M	73	Metastatic sarcomatoid carcinoma of lymph nodes	200 mg once, once every 3 weeks	NA	10	Dry mouth, polydipsia, polyuria, dizziness, chest tightness, nausea, vomiting, fatigue, weight loss	99.1	7.8
4	Zhang et al. (2024)	F	74	Left ureteral carcinoma	200 mg once, once every 3 weeks	NA	5	Thirst, lethargy	36.93	7.9
5	Wang Y. Z. et al. (2024)	F	63	Esophageal carcinoma	200 mg once, once every 3 weeks	Amlodipine, metoprolol	4	Fatigue, nausea	33.7	NA
6	Wang L. et al. (2024)	F	78	Lung adenocarcinoma	200 mg once	Bevacizumab	4	Dry mouth, polydipsia, polyuria, lethargy, breath smells of rotten apples	26.19	8.4
7	Pang et al. (2024)	M	79	Renal clear cell carcinoma with retroperitoneal lymph node metastasis	200 mg once, once every 3 weeks	Kangai injection, lentinan for injection	13	Thirst, polydipsia, polyuria, poor appetite, fatigue, nausea, vomiting	35.8	8.3
8	Yang et al. (2023)	M	54	Gastric malignant tumor	NA	Docetaxel + cisplatin for 1 cycle, followed by paclitaxel for injection (albumin bound) for 7 cycles	8	Dry mouth, polydipsia, polyuria, blurred vision, slow response, blurred mind	18.8	10.2
9	Yang et al. (2023)	M	61	Intrahepatic cholangiocarcinoma	NA	Fluorouracil + oxaliplatin + lenvatinib	2	Dry mouth, polydipsia, polyuria, emaciation	11.3	9.1
10	Kong et al. (2023)	M	63	Stage IV gastric cancer	NA	Oxaliplatin + capecitabine for 6 cycles, followed by capecitabine	15	Thirst, polyuria	43.11	9.8
11	Mei et al. (2024)	M	73	Cardiac cancer	200 mg once	Trastuzumab	2	Dizziness, fatigue, dry mouth, polydipsia, polyuria, poor feeding	52.4	8.8
12	Mei et al. (2024)	M	60	Lung squamous cell carcinoma	200 mg once	Paclitaxel for injection (albumin bound)	6	Sudden chest tightness, shortness of breath	37.2	NA
13	Wang Y. et al. (2024)	NA	NA	NA	200 mg once, once every 3 weeks	NA	3	NA	23.84	NA
14	Wang Y. et al. (2024)	NA	NA	NA	200 mg once, once every 3 weeks	NA	14	NA	26.68	NA
15	Han et al. (2023)	M	65	Right ureteral squamous cell carcinoma	NA	NA	7	NA	64	7.7
16	Qiu et al. (2023)	M	56	Gastric cancer	NA	NA	16	Dry mouth, polydipsia, polyuria	18.7	8.2
17	Our case	M	54	Renal transitional cell carcinoma	200 mg once, once every 3 weeks	Telmisartan, aspirin, rosuvastatin	11	Dry mouth, polydipsia, polyuria	21.37	7.2

Patient	Authors, Y	Fast serum C-peptide at the first visit (ng/mL)	Pancreatic autoantibodies	DKA	Other endocrine adverse events	CTCAE 5.0	Discontinue tislelizumab	Therapy	Outcome	Reassessment of causality
1	Kong et al. (2023)	0.17	Negative	Yes	No	Grade 4	NA	Preprandial insulin 3 times a day + basal insulin, ih	Well controlled glycaemia, long-term insulin needs	Probable
2	Zhang et al. (2022)	0.175 (0.098, at reexamination)	Negative	Yes	Subclinical hypothyroidism, autoimmune thyroiditis	Grade 3	NA	Preprandial insulin 3 times a day + basal insulin, ih	Greatly fluctuated glycaemia, long-term insulin needs	Probable
3	Rui and Zhu (2023)	0.06 (0, at reexamination)	NA	Yes	No	Grade 4	No	Preprandial insulin 3 times a day + basal insulin, ih	Well controlled glycaemia, long-term insulin needs	Probable
4	Zhang et al. (2024)	0.27	Negative	Yes	No	Grade 4	Yes	Preprandial insulin 3 times a day + basal insulin, ih	Well controlled glycaemia, but termination of follow-up due to tumor progression	Probable
5	Wang et al. (2024)	0.44 (0.01, at reexamination)	Negative	Yes	No	Grade 4	Yes	Insulin therapy	Poorly controlled glycaemia, and transferred for further treatment	Probable
6	wang et al., 2024	0.1	Negative	Yes	Hypothyroidism	Grade 3	No	Insulin therapy	Well controlled glycaemia, long-term insulin needs	Probable
7	Pang et al. (2024)	0.19	Negative	Yes	Hypothyroidism	Grade 4	Yes	Preprandial insulin 3 times a day + basal insulin, ih	Well controlled glycaemia, long-term insulin needs	Probable
8	Yang et al. (2023)	NA	Negative	Yes	Hypophysitis	Grade 3	No	Insulin therapy	Well controlled glycaemia, but die of tumor progression	Probable
9	Yang et al. (2023)	0.57	Negative	Yes	No	Grade 2	No	Insulin therapy	Well controlled glycaemia, long-term insulin needs	Probable
10	Yang et al. (2023)	0.19	Negative	No	No	Grade 4	Yes (discontine after 4 cycles)	Insulin, ih; Metformin, po	Poorly controlled glycaemia, but die of tumor progression	Probable
11	Mei et al. (2024)	NA	NA	Yes	No	Grade 4	Yes (discontine after 1 cycle)	Preprandial insulin 3 times a day + basal insulin, ih	Poorly controlled glycaemia, and transferred for further treatment	Definite
12	Mei et al. (2024)	NA	NA	Yes	No	Grade 4	Yes	Insulin, q12h ih; Prednisone, dapagliflozin	Well controlled glycaemia, but die of tumor progression	Probable
13	Wang et al. (2024)	0.09	Negative	No	No	Grade 3	Yes	Insulin therapy	Generally controlled glycaemia, long-term insulin needs	Probable
14	Wang et al. (2024)	0.08	Negative	No	No	Grade 3	Yes	Insulin therapy	Generally controlled glycaemia, long-term insulin needs	Probable
15	Han et al. (2023)	0.047	Negative	NA	No	Grade 4	No	Insulin therapy	Greatly fluctuated glycaemia, long-term insulin needs	Probable
16	Qiu et al. (2023)	0.27	Negative	NA	No	Grade 3	NA	Insulin therapy	Glycaemia: NA, long-term insulin needs	Probable
17	Our case	<0.02	Negative	Yes	No	Grade 3	Yes	Preprandial insulin 3 times a day + basal insulin, ih; Metformin, acarbose, linagliptin, dorzagliatin, po	Generally controlled glycaemia, long-term insulin needs	Probable

DKA, diabetic ketoacidosis; CTCAE, 5.0, Common Terminology Criteria for Adverse Events Version 5.0; NA, not available.

### 3.2 Incidence of tislelizumab-induced IDDM

The overall frequency of IDDM as an endocrine immune-related adverse event is relatively low (<1%); however, this event has high clinical significance (Stamatouli et al., 2018). Nearly all the reported cases of ICI-induced IDDM have been attributed to anti-PD-1 therapies, including pembrolizumab and nivolumab. Several cases of anti-PD-L1 therapy have been reported. ICI-induced IDDM appears extremely rare following anti-CTLA-4 monotherapy (Chang et al., 2019). However, to date, no reports of tislelizumab-induced IDDM have been published in PubMed. Only 16 cases of tislelizumab-related IDDM with relatively complete data were found in Chinese article databases. This indicated a very low incidence of tislelizumab-induced IDDM, which is consistent with previous reports (Stamatouli et al., 2018; Kotwal et al., 2019; Tsang et al., 2019).

### 3.3 Clinical characteristics of tislelizumab-induced IDDM

In the 15 reported cases, mean age was 65.7 years old (range, 54–79 years), consistent with the previous reports (Stamatouli et al., 2018; Kotwal et al., 2019; Byun et al., 2020; Clotman et al., 2018; Baden et al., 2018; de Filette et al., 2019). This is clearly different from the classic type 1 diabetes mellitus (T1DM), which is common in children and adolescents. 73% (11/15) of the cases were male, higher than the previously reported ratio of approximately 60% (Stamatouli et al., 2018; Byun et al., 2020; Kotwal et al., 2019; Baden et al., 2018), trending towards the previously reported 90% ratio (Qiu et al., 2023), which may be due to the limited number of cases, the presence of tumors in the sex difference, or ethnic differences.

Among these 15 patients, six (40%) had digestive system tumors, five (33.3%) had urinary system tumors, three (20%) had NSCLC, and one (6.7%) had metastatic sarcomatoid carcinoma of the lymph nodes. Nine of the 17 patients (53%) received drug combination treatment, and eight of the 17 patients (47%) had no description in the original literature, perhaps tislelizumab monotherapy (Table 2). Among the 17 patients, the mean duration until diabetes onset after initiating tislelizumab treatment was after 8.4 cycles, within 6 months of treatment, similar to previous reports (Chang et al., 2019; Byun et al., 2020; Kotwal et al., 2019; Baden et al., 2018; de Filette et al., 2019; Okamoto et al., 2016). The longest duration was after 18 cycles, which showed the latent period may be long, and clinicians should avoid ignoring tislelizumab-induced IDDM.

Of the 17 reported cases, one presented with subclinical hypothyroidism and autoimmune thyroiditis (Zhang et al., 2022), two with hypothyroidism (Wang L. et al., 2024; Pang et al., 2024), and one with hypophysitis (Yang et al., 2023) (Table 2), showing tislelizumab may simultaneously induce several endocrine immune-related adverse events.

Based on the available reported cases, 80% of patients presented with DKA, with a high mean (range) random blood glucose of 35.9 (11.3–99.1) mmol/L. The average (range) HbA1c value was 8.5% (7.2%–10.2%) at diagnosis, indicating that some degree of hyperglycemia was present prior to the acute presentation. In the reported 14 patients, the average (range) serum levels of C-peptide was 0.19 ng/mL (<0.02–0.57 ng/mL). Reexamination revealed absent or inappropriately low serum C-peptide levels in three of the 14 patients (Zhang et al., 2022; Rui and Zhu, 2023; Wang Y. Z. et al., 2024). These data demonstrated a rapid loss of *β*-cell function accompanied by acute progression to hyperglycemia. These clinical and laboratory features were consistent with previous reports of ICI-induced IDDM (Stamatouli et al., 2018; Byun et al., 2020; Kotwal et al., 2019; Clotman et al., 2018; Baden et al., 2018; Gauci et al., 2017; de Filette et al., 2019; Okamoto et al., 2016).

However, 100% (14/14) of the reported cases exhibited undetectable islet autoantibodies, including ICA, IAA, anti-GAD antibody, anti-IA-2 antibody, and anti-ZnT8 antibody, which differs from previous reports on ICI-induced IDDM (Stamatouli, et al., 2018; Byun et al., 2020; Kotwal et al., 2019; Clotman et al., 2018; Gauci et al., 2017; de Filette et al., 2019). De Filette et al. (de Filette et al., 2019) demonstrated that at least one islet autoantibody was positive in 53% (47/88) of the analyzed patients with ICI-induced T1DM, while the GAD antibody was the most positive in 51% of patients. The ethnicity was Asian in 15% of the study (de Filette et al., 2019). However, the rate of islet autoantibody positivity in Japanese patients with ICI-induced T1DM is lower than that in Caucasians (4.76% vs. 56.00%) (Baden et al., 2018). In addition, only one (10%) of 10 Chinese patients with ICI-induced T1DM was anti-GAD antibody-positive (Qiu et al., 2023). This finding suggests that pancreatic autoantibodies against ICI-induced IDDM was almost absent in East Asians, which may be due to racial differences. Furthermore, we found that several recent studies supported our hypothesis. Qiu et al. (Qiu et al., 2022) showed that islet autoantibody positive patients with ICI-induced T1DM had prominently higher prevalence in Caucasians than in Asians (45.7% vs. 9.5%), and had higher proportion of human leukocyte antigen (HLA) susceptibility alleles for T1DM than islet autoantibody negative patients (88.9% vs. 44.0%). Clinical studies showed that the susceptible HLA-DR4 haplotypes were less frequent in Chinese patients with ICI-induced T1DM than in Caucasians (2.3% vs. 76%) (Liu et al., 2023; Stamatouli et al., 2018). Additionally, the small number of cases and characteristics of tislelizumab were not excluded.

We found that previous related reports were named “PD-1 inhibitor or ICI-induced T1DM” (Clotman et al., 2018; Baden et al., 2018; de Filette et al., 2019) or “fulminant T1DM” (Okamoto et al., 2016; Kong et al., 2023; Rui and Zhu, 2023; Zhang et al., 2024) or “PD-1 inhibitor or ICI-induced IDDM” (Stamatouli et al., 2018; Kotwal et al., 2019). Fulminant T1DM is a subtype of T1DM that was first described in Japan (Imagawa et al., 2000). In 2012, the Japan Diabetes Society showed that fulminant T1DM is diagnosed when all the following three findings are present: (1) occurrence of diabetic ketosis or ketoacidosis soon (about 7 days) after the onset of hyperglycemic symptoms (elevation of urinary and/or serum ketones at first visit), (2) plasma glucose ≥288 mg/dL and HbA1c < 8.7% at first visit, and (3) urinary C-peptide excretion <10 ug/d or fasting serum C-peptide level <0.3 ng/mL and serum C-peptide <0.5 ng/mL after intravenous glucagon (or after a meal) at onset (Imagawa et al., 2012). ICI-induced IDDM, as in our case, has many clinical features similar to those of fulminant T1DM. In addition, islet autoantibodies were generally undetectable in both patients with fulminant T1DM and the cases included in this study.

Notably, several findings differed between the patients with fulminant T1DM and those with ICI-induced IDDM. The age of onset was >20 years in adults with fulminant T1DM but >60 years in those with ICI-induced IDDM (Chang et al., 2019; Imagawa et al., 2000). The inducing factors are involved in drug hypersensitivity, viral infections, pregnancy et al. in fulminant T1DM, but ICIs are involved in ICI-induced IDDM (Chang et al., 2019; Imagawa et al., 2000). The time of disease onset is usually within 1 week in fulminant T1DM but within 3 months in ICI-induced IDDM (Chang et al., 2019; Imagawa et al., 2000). ICI-induced IDDM is similar to but not fulminant T1DM, which is obviously different from classic T1DM. Therefore, we believe that “PD-1 inhibitors, ICI-induced IDDM” are relatively accurate nomenclatures and was used in our case report.

### 3.4 Therapy of tislelizumab-induced IDDM

When these patients developed hyperglycemia, they immediately received subcutaneous insulin injections and symptomatic treatment. All of our reported patients with available data had a long-term need for insulin, which is consistent with previous reports (Chang et al., 2019; Stamatouli et al., 2018; Byun et al., 2020; Kotwal et al., 2019; Clotman et al., 2018; Baden et al., 2018; Gauci et al., 2017; de Filette et al., 2019).

According to CTCAE5.0 of United States in 2017, the severity criteria of adverse events about diabetes was as follows: (1) Grading1: Asymptomatic or mild symptoms; fasting glucose value > upper limit of normal; fasting glucose value <8.9 mmol/L; no evidence of ketosis or laboratory evidence of T1DM; (2) G2: Moderate symptoms, able to perform activities of daily living (ADL), fasting glucose value is 8.9–13.9 mmol/L, ketosis or evidence of T1DM at any glucose level; (3) G3-4: Severe symptoms, medically significant or life-threatening outcomes, unable to perform ADL; fasting glucose value of G3 is 13.9–27.8 mmol/L, G4 > 27.8 mmol/L (Freites-Martinez et al., 2021; Brahmer et al., 2018). The occurrence of ICI-induced IDDM is not a contraindication for continuing ICIs, and patients can continue ICIs with close clinical follow-up and laboratory evaluations. Patients with G2 or higher may hold ICIs until glucose control is achieved with a reduction in toxicity to G1 or less (Brahmer et al., 2018). Among the 17 cases with available CTCAE5.0, nine (52.9%) presented with grade 4, seven (41.2%) with grade 3, and one (5.9%) with grade 2. All these cases were grade 2 or higher and tislelizumab should be discontinued. However, only 50% (7/14) of the patients discontinued tislelizumab immediately, 7.1% (1/14) discontinued tislelizumab after four cycles, and 7.1% (1/14) discontinued after one cycle. Therefore, real-world therapy is looser than these guidelines are.

At the end of follow-up, eight (50%) of the 16 patients that reported these data had relatively stable glycemic control. Three (21.4%) of 14 patients with available information died of tumor progression (Yang et al., 2023; Mei et al., 2024) (Table 2). Unlike other endocrine adverse events, corticosteroids do not appear to play a role in the treatment of ICI-induced IDDM, although evidence remains extremely limited. Four patients with ICI-induced IDDM were treated with systemic corticosteroids, and none were successful in reversing the ICI-induced IDDM (Lowe et al., 2016; Chae et al., 2017; Smith-Cohn et al., 2017; Aleksova et al., 2016).

### 3.5 Risk factors

ICI-induced IDDM is associated with genetic susceptibility. Stamatouli et al. at Yale University described 27 patients with ICI-induced IDDM, and identified HLA genotypes in 23 cases (Stamatouli et al., 2018). There was a predominance of HLA-DR4, which was present in 76% (16/21) of the patients and was significantly higher than the reported frequencies in American Caucasians (17.3%) and even in patients with spontaneous T1DM (Stamatouli et al., 2018; Erlich et al., 2008). However, other spontaneous T1DM high-risk alleles were not overrepresented, including HLA-DR3, -DQ2, and -DQ8 (Erlich et al., 2008; Stamatouli et al., 2018).

### 3.6 Pathogenesis

PD-1 is generally expressed on chronically activated T cells in peripheral tissues, particularly CD^8+^ T cells. By binding to its ligands, PD-L1 and PD-L2, which are expressed on stromal cells, tumor cells, and antigen-presenting cells, PD-1 transmits negative signaling events in such T cells and induces their apoptosis of T cells (Bour-Jordan et al., 2011). Pancreatic *β*-cells express PD-L1, which evades the immune response. Anti-PD-1 antibodies improve survival by activating T cells to restore antitumor immunity. However, normal tissues, such as pancreatic *β*-cells, may be affected by activated T-cells, leading to immune-related adverse events such as PD-1 inhibitor-related diabetes (Clotman et al., 2018). Animal experiments (Ansari et al., 2003) and clinical studies (Li et al., 2020; Yoneda et al., 2019; Colli et al., 2018) have provided relevant evidence.

ICIs cause pancreatic damage (Byun et al., 2020; Kotwal et al., 2019). Therefore, ICI-induced IDDM is mostly insulin-deficient, and the underlying mechanism appears similar to that of classic T1DM. However, compared with classic T1D, the islet function impairment in ICI-induced IDDM patients is more rapid and significant, similar to fulminant T1DM (Chang et al., 2019; Imagawa et al., 2012). ICI-induced IDDM is a special type of DM that differs from classic T1DM and fulminant T1DM.

## 4 Conclusion

To summarize, this is the first study to report a case of tislelizumab-induced IDDM, accompanied by a literature review, enabling the characterization of tislelizumab-induced IDDM resulting from treatment toxicity. Similar to previous reports, it is characterized by a faster progression to severe insulin deficiency than classic T1DM, frequently presents with DKA, and needs exogenous insulin for long time. However, in contrast to previous reports on ICI-induced IDDM in Western countries, our reported Chinese cases were negative for islet autoantibodies, possibly because of racial differences. As immunotherapies have become more prevalent, the case number of ICI-induced IDDM has increased. Better characterization of ICI-induced IDDM will provide references for the clinical identification, treatment, and reduction of the risk of this adverse reaction. Considering the potential severity of ICI-induced IDDM with the frequent onset of DKA, patients should be motivated to monitor glycemia during immunotherapy.

## Data Availability

The original contributions of this study are included in the article. Further inquiries can be directed to the corresponding author.

## Abbreviations

ICIs, immune checkpoint inhibitors; CTLA-4, cytotoxic T lymphocyte antigen-4; PD-1, programmed cell death protein 1; PD-L1, programmed death-ligand 1; HL, Hodgkin lymphoma; NMPA, National Medical Products Administration; EMA, European Medicines Agency; USFDA, U.S. Food and Drug Administration; NSCLC, non-small cell lung cancer; IDDM, insulin-dependent diabetes mellitus; HbA1c, hemoglobin A1c; DKA, diabetic ketoacidosis; CTCAE5.0, Common Terminology Criteria for Adverse Events 5.0; DM, diabetes mellitus; ICA, islet cell antibodies; IAA, insulin autoantibodies; GAD, glutamic acid decarboxylase; IA-2, insulinoma-associated antigen-2; ZnT8, zinc transporter 8; CSII, continuous subcutaneous insulin infusion; T1DM, type 1 diabetes mellitus; ADL, activities of daily living; HLA, human leukocyte antigen.
